# Oligopeptides as full-length New Delhi metallo-β-lactamase-1 (NDM-1) inhibitors

**DOI:** 10.1371/journal.pone.0177293

**Published:** 2017-05-23

**Authors:** Bingzheng Shen, Chengliang Zhu, Xiang Gao, Gang Liu, Jinchun Song, Yan Yu

**Affiliations:** 1 Department of Pharmacy, Renmin Hospital, Wuhan University, Wuhan, China; 2 State Key Laboratory of Virology, Wuhan University, Wuhan, China; 3 Department of Clinical Laboratory, Renmin Hospital, Wuhan University, Wuhan, China; 4 Central Laboratory, Renmin Hospital, Wuhan University, Wuhan, China; 5 Department of Gastroenterology, Tongji Hospital, Tongji Medical College, Huazhong University of Science and Technology, Wuhan, China; Kaohsiung Medical University, TAIWAN

## Abstract

‘Superbug’ bacteria producing NDM-1 enzyme causing wide public concern were first detected in a patient who visited India in 2008. It's an effective approach to combining β-lactam antibiotics with NDM-1 inhibitor for treating NDM-1 producing strain infection. In our research, we designed ten oligopeptides, tested IC_50_ values against NDM-1 enzyme, determined the MIC values of synergistic antibacterial effect and explored the binding model. We found that the oligopeptides 2 (Cys-Phe) and 5 (Cys-Asp) respectively presented IC_50_ values of 113 μM and 68 μM and also displayed favorable synergistic effects of the inhibitors in combination with ertapenem against genetic engineering-host *E*. *coli* BL21 (DE3)/pET30a-NDM-1 and a clinical isolate of *P*. *aeruginosa* with *bla*_NDM-1_. Flexible docking and partial charge study suggested the interaction between oligopeptide and NDM-1. Three types of action effects, hydrogen bond, electrostatic effect and π-π interaction, contributed to the inhibitory activities.

## Introduction

Since penicillin was used in the treatment of bacterial infections, humans have always struggled against antimicrobial-resistant bacteria. It is the fact that many patients have died because of no effective anti-infection drugs [1–3]. For the majority of clinical drug resistant bacteria, there are four main biochemical types of mechanisms [4]: 1 antibiotic inactivation; 2 target modification; 3 altered permeability; 4 “bypass” metabolic pathway. Producing β-lactamase is one of the ways that bacteria inactivate β-lactam antibiotics. It is known about 300 β-lactamases mostly produced by gram negative bacteria [4,5]. According to the classification system of Ambler, these β-lactamases have been comprised four classes: A, B, C and D [6,7]. B class β-lactamases are also called metallo-β-lactamases (MβLs), in which one or two zinc ions are necessary for catalytic activity. On the basis of the known sequences, this kind of β-lactamases (B class) has been further classified into three subclasses B1, B2 and B3 [7].

New Delhi metallo-β-lactmase-1 (NDM-1) was initially reported in *Klebsiella pneumoniae* from India belonging to the subclass B1 MβLs superfamily [8]. To date, bacteria carrying *bla*_NDM-1_ genes have appeared throughout the world [9]. Recently NDM-1 producing bacteria from animals reported more frequently [10–12], because the water polluted with excrement or human waste played a crucial role for animals to acquire antimicrobial-resistant bacteria [13]. NDM-1 is a single-chain hydrolytic enzyme composed of 270 amino acids and two zinc ions in the core area, which N-terminal has a type I lipidation signal peptide domain appearing to be correlation with guiding the mature protein to the bacterial outer membrane [14]. The crystal structures of NDM-1 and hydrolysis mechanism revealed the key catalytic domain and the characteristics of enzymatic hydrolysis of the β-lactam ring in the structure of β-lactam antibiotics. Like most other MβLs, NDM-1 presents representative sandwich structure of αβ/βα fold and five α-helices on its external surface. The catalytic center of NDM-1 flanked by two important loops (Loop 3 and Loop 10) is located at the bottom. Two zinc ions, one bridging water and six key amino acids played a major role in hydrolytic activity. Zn1 is coordinated by three conserved histidine residues (His120, His122 and His189) and a bridging water (W1), and Zn2 is coordinated by three unaltered amino acids (Asp124, Cys208 and His250) [15–17].

In spite of the urgency for treatment of NDM-1 expressing bacteria, there has no much progress on exploring inhibitors for clinical use.

Based on the biochemical characterization of NDM-1 enzyme and molecular models of enzyme active center in the catalytic domain, oligopeptides were discovered to inhibit the hydrolytic activity of NDM-1, and the half maximal inhibitory concentration (IC_50_) was determined. Synergistic effect was also confirmed through combining these oligopeptides with ertapenem against genetically engineered bacterium producing NDM-1 and a clinical isolate of *P*. *aeruginosa* with *bla*_NDM-1_. The binding model suggested that hydrogen bond, electrostatic interaction between inhibitor and zinc ion, as well as π-π interaction in the active site of the enzyme played important roles in bioactivities.

## Materials and methods

### Testing antibiotic susceptibility

*E*. *coli* BL21(DE3) cells were transformed with the pET30a plasmid encoding the NDM-1 gene (Gen Bank ID: **HQ162469**). *E*. *coli* BL21(DE3)/pET30a-NDM-1 was grown overnight at 37°C in liquid Luria-Bertani (LB) broth containing 50μg/ml kanamycin, 100μg/ml ampicillin and 50μM Zn(NO_3_)_2_ to select for *E*. *coli* clones containing the plasmid of pET30a-NDM-1. Two strains, *E*. *coli* BL21 (DE3)/pET30a-NDM-1 overexpressing NDM-1 and *P*. *aeruginosa* isolated from Renmin Hospital of Wuhan University with *bla*_NDM-1_ confirmed by PCR (data not shown), were cultured overnight, respectively. The cultures were diluted 1:100, grown to an optical density at 600 nm (OD_600_) of ~0.6, and spread onto LB agar. After naturally drying for five minutes, E-test strips (AB Biodisk, Solna, Sweden) were applied to the ~150mm Mueller-Hinton (MH) (Tianhe Microbial Agents, Hangzhou, China) agar plates and grown for 24h at 35°C to 37°C without further intervention. The antibiotic strips respectively containing penicillin G, ampicillin, cephalothin, cefuroxime, ceftazidime, cefepime, meropenem, ertapenem, aztreonam were used for testing susceptibility. The results were obtained in accordance with the Clinical and Laboratory Standards Institute (CLSI) guidelines [18].

### Determining IC_50_ values for inhibitors

The full-length NDM-1 enzyme was expressed and purified by a method from a previously published procedure [19]. All the oligopeptides were synthesized by GL Biochem. (Shanghai), Ltd., China, with a purity of >95%. The target of NDM-1 enzyme was pre-incubated with the desired oligopeptide for 15min at 35°C before the assay by adding the inhibitor. The oligopeptides used for testing were dissolved in 10mM phosphate-buffered saline (PBS) buffer (pH7.0), containing dimethyl sulfoxide (DMSO) with the final concentration of 0.25% (V/V). No effect of DMSO was observed on the enzyme activity at this concentration. Ertapenem (80μM) was used as report substrate. The hydrolysis of the substrate by the NDM-1 was detected by monitoring the variation in absorbance resulting from the opening of the β-lactam ring. All the determinations were made on a spectrophotometer (UV-1750, Shimadzu, Japan) connected to a personal computer. The enzymatic reactions were carried out in a total volume of 100μL. The reaction buffer contained 20mM HEPES buffer, pH7.3, 0.25M NaCl, 1mM 1,4-Dithio-DL-threitol (DTT) (Beinglay Biotech Corp., Wuhan, China), 50μM Zn(NO_3_)_2_ and 10mg/ml bovine serum albumin (BSA) preventing NDM-1 denaturation [20]. The ultraviolet (UV) absorbance detector was set at 300 nm, a λ _max_ for ertapenem [21,22]. The IC_50_ values were determined more than three times by fitting the concentration dependence of residual enzyme activity to the nonlinear regression (curve fit) using GraphPad Prism (GraphPad Software, La Jolla, America).

### Evaluating synergy effect of β-lactam antibiotic combined with oligopeptide

*E*. *coli* BL21 (DE3)/pET30a-NDM-1 overexpressing NDM-1 and a clinical isolate of *P*. *aeruginosa* with *bla*_NDM-1_ were also utilized to evaluate the bioactivities of oligopeptides, which reversed NDM-1-mediated β-lactam antibiotics resistance. Determination of antibacterial synergy was performed using a serial twofold dilutions method recommended by CLSI guidelines [18]. *E*. *coli* BL21 (DE3)/pET30a was used as a control in the experiment. Minimum inhibitory concentrations (MICs) of the agents were determined by the twofold serial dilution with Mueller-Hinton (MH) broth (Shifeng Biotech Corp., Shanghai, China). For this assay, *E*. *coli* BL21 (DE3)/pET30a-NDM-1 was grown overnight in MH broth media containing 50μg/ml kanamycin at 37°C [23]. β-lactam antibiotic and oligopeptide at various concentrations were added to 120μL broth media of a 1:20 dilution of the overnight culture, resulting the final volume of 150μL [24]. The growth of bacterial cultures was set up in 96-well microplates, which incubated at 37°C with constant shaking continuously for 18 h and a reading at 630 nm was taken to determine the MICs of synergistic effect [25]. The lowest antibiotic concentration preventing visible growth was deemed to be the MIC value. To validate whether oligopeptides possess antibiotic properties, the oligopeptides were tested without β-lactam antibiotics at various concentrations with *E*. *coli* BL21 (DE3)/pET30a-NDM-1. Every data of reading was repeated thrice, and each experiment mentioned above was performed at least two times.

### Binding model research for interaction between oligopeptide and NDM-1

Molecular docking is a commonly used and effective method in the research of binding model and structure-activity relationship to predict the interaction pattern between ligand and the target of known 3D-structure. Considering that the catalytic center was at the bottom of NDM-1 and many loops around it, flexible docking integrated in Discovery Studio version2.5 (DS v2.5) (Accelrys Inc., San Diego, CA) was carried out to study binding model, which oligopeptides can be performed to force the target into alternative conformations [26]. NDM-1 complexes with substrate (PDB code: 3Q6X) were selected from Protein Data Bank (PDB) for docking. Hydrolyzed ampicillin was removed from the crystal structure. 10Å around the center of di-zinc ions was defined as ligand-binding domain. This domain completely covered the original area of hydrolyzed ampicillin. Water molecules in the binding domain were retained, because water-bridge and two zinc ions served as the important electron transfer medium in the process of breaking β-lactam rings [15, 16]. Zn1 was coordinated by His120, His122 and His189, as well as Zn2 was coordinated by Asp124, Cys208 and His250. The seven key amino acids, six amino acids binding two di-zinc ions and Tyr229, were treated as flexible residues [27]. These amino acids were allowed to create plenty of flexible conformations in the process of flexible docking. For the oligopeptide ligands, energy minimization method was carried out, which based on the energy of a structure through geometry optimization. Accelrys CHARMm force field [28] was conducted throughout the simulation, and all other parameters were set to default values in the program. The MM-PBSA method, a popular method for calculating binding affinities of biomolecular complexes, was used to calculate the binding free energy.

## Results and discussion

### Susceptibility results by E-test method

The antimicrobial susceptibility patterns of *E*. *coli* BL21 (DE3)/pET30a-NDM-1, *E*. *coli* BL21 (DE3)/pET30a, *E*. *coli* BL21 *and P*. *aeruginosa* with *bla*_NDM-1_ are presented in Table 1. The bacteria of *E*. *coli* BL21 (DE3)/pET30a and *E*. *coli* BL21(DE3) displayed the similar β-lactam antibiotic susceptibility, indicating that whether carrying an empty vector (pET30a) had no any effect on susceptibility of the host.

**Table 1:** MIC values of β-lactam antibiotics determined by the E-test method at 24 h.

Antibiotic	MIC (μg/ml)			
	*E*. *coli* BL21 (DE3)/ pET30a-NDM-1	*E*. *coli* BL21 (DE3)/ pET30a	*E*. *coli* BL21 (DE3)	*P*. *aeruginosa* with *bla*_NDM-1_
**Penicillins**				
Penicillin G	>32	12	12	>32
Ampicillin	>256	8	6	>256
Piperacillin	ND ^a^	ND	ND	ND
**Cephalosporins**				
Cephalothin	>256	4	6	>256
Cefoxitin	ND	ND	ND	ND
Cefotaxime	>256	0.25	0.125	>256
Cefuroxime	>256	3	2	256
Ceftazidime	>256	0.25	0.38	>256
Cefepime	>64	0.08	0.08	>64
**Carbapenems**				
Meropenem	>32	0.047	0.032	>32
Ertapenem	>32	0.094	0.094	>32
**Monobactam**				
Aztreonam	>256	0.064	0.047	>256

^a^ ND, not determined.

*E*. *coli* BL21 (DE3)/pET30a-NDM-1 and *P*. *aeruginosa* with *bla*_NDM-1_ showed a decrease in susceptibility to penicillin G, ampicillin, cephalothin, cefuroxime, ceftazidime, cefepime, meropenem, ertapenem and aztreonam, demonstrating that the NDM-1 MβL can lead to β-lactam antibiotic resistance. The antimicrobial susceptibility pattern of the genetically engineered bacterium of *E*. *coli* BL21 (DE3)/pET30a-NDM-1 was also found to be similar to the clinical isolate of *P*. *aeruginosa* with *bla*_NDM-1_, with the MIC more than the maximum values of E-test antibiotic strips.

### IC_50_ values and structure-activity relationship of oligopeptides against full-length NDM-1

**Fig 1:**
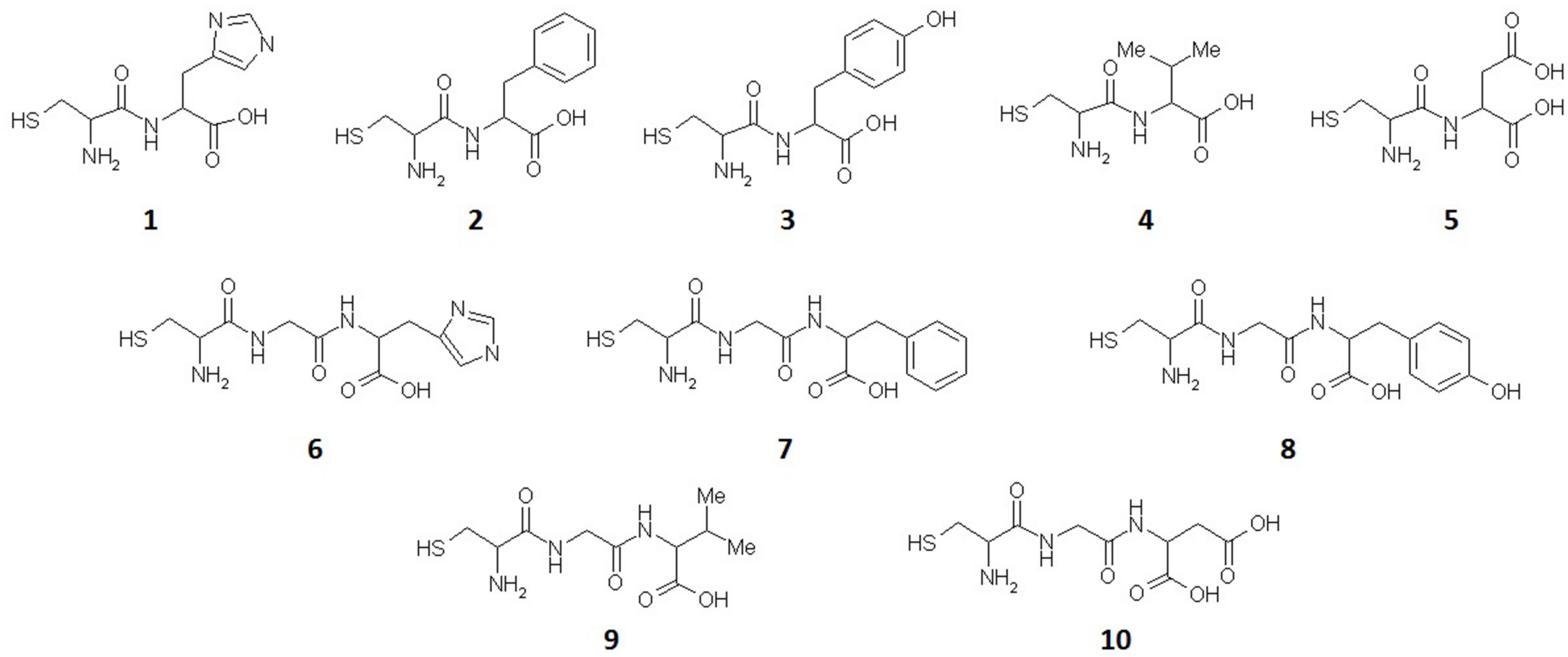
Molecular structure of all oligopeptides against NDM-1.

The carbapenem antibiotic, ertapenem, was used as report substrate to determine the inhibitory activities of oligopeptides. According to the determination method mentioned above, all the tests repeated more than thrice. As shown in Fig 1 and Table 2, the results indicated that most of the oligopeptides had inhibitory effect of varying degrees respectively. Oligopeptides 2 (Cys-Phe), 5 (Cys-Asp) and 7 (Cys-Gly-Phe) presented better inhibitory activities. The IC_50_ values were 113 μM, 68 μM and 144 μM, respectively. Oligopeptides 1 (Cys-His), 4 (Cys-Val), 6 (Cys-Gly-His) and 9 (Cys-Gly-Val) showed relatively weak NDM-1 inhibitory activities at a concentration range of 150–300 μM. Oligopeptides 3 (Cys-Tyr), 8 (Cys-Gly-Tyr) and 10 (Cys-Gly-Asp) displayed no obvious inhibitory effect below a concentration of 300 μM.

**Table 2:** Inhibitory activities of oligopeptides against full-length NDM-1.

Oligopeptides	Amino acid sequences ^a^	IC_50_ (μM) (Mean±SD)
**Di-peptide**		
1	Cys-His	182±14
2	Cys-Phe	113±9
3	Cys-Tyr	>300
4	Cys-Val	235±18
5	Cys-Asp	68±5
**Tri-peptide**		
6	Cys-Gly-His	263±21
7	Cys-Gly-Phe	144±13
8	Cys-Gly-Tyr	>300
9	Cys-Gly-Val	249±21
10	Cys-Gly-Asp	>300

^a^ All the residues in oligopeptides are natural amino acids (L-amino acid).

Oligopeptide 5 containing the substructure of succinic acid and 3-mercaptopropionic acid had the best activity in all testing samples (IC_50_ = 68 μM). Comparing the structures of two oligopeptides 5 and 10, the difference is a glycine inserted in the middle. The increase of the glycine might lead to the loss of the inhibitory activity (IC_50_>300 μM). When other di-peptides 1 (IC_50_ = 182 μM), 2 (IC_50_ = 113 μM) and 4 (IC_50_ = 235 μM) were contrasted with the corresponding tri-peptides 6 (IC_50_ = 263 μM), 7 (IC_50_ = 144 μM) and 9 (IC_50_ = 249 μM), IC_50_ values indicated that the inhibitory activities appeared varying degrees of decline with one glycine inserted.

The micro-structural differences between oligopeptides 2 and 3, the hydroxyl of benzyl group at the functional side chain, caused the huge difference of inhibitory effect (IC_50_ = 113 and >300 μM, respectively). The IC_50_ value of oligopeptide 4, which benzyl group of oligopeptide 2 was replaced by iso-propyl, displayed approximately a two-fold IC_50_ value of oligopeptide 2 (IC_50_ = 235 and 113 μM, respectively). Analyzing the structure-activity relationship of oligopeptides 2–4, we found that the inhibitory effects against NDM-1 enzyme were positive correlation with the hydrophobic effect of side chain (hydrophobicity: benzyl group of oligopeptide 2 > iso-propyl group of oligopeptide 4 > *p*-hydroxybenzyl group of oligopeptide 3).

### Synergistic antibacterial effect of ertapenem in combination with oligopeptide

*E*. *coli* BL21 (DE3)/pET30a-NDM-1 overexpressing NDM-1 and *P*. *aeruginosa* with *bla*_NDM-1_ displayed high-level resistance to ertapenem. The strain was still able to grow in the broth media containing ertapenem at a concentration of 256 μg/ml. Oligopeptides 1, 2, 5 and 7, possessing IC_50_< 200μM, were chosen as representative inhibitors for determining synergistic antibacterial effects with ertapenem. None of the oligopeptides possessed any antibacterial activity within the concentration range of 25–200 μM. The MIC values of ertapenem in combination with inhibitory oligopeptides at various concentrations against *E*. *coli* BL21 (DE3)/pET30a-NDM-1 and the clinical isolate of *P*. *aeruginosa* were presented in Table 3.

**Table 3:** Synergistic antibacterial activity of inhibitory oligopeptides in combination with ertapenem against genetically engineered bacterium of *E*. *coli* BL21 (DE3)/pET30a-NDM-1 and a clinical isolate of *P*. *aeruginosa* ^a^.

Oligopeptides	Amino acid sequences	MICs (μg/ml) of ertapenem in combination with oligopeptides at various concentrations							
		*E*. *coli* BL21 (DE3)/pET30a-NDM-1				Clinical isolate of *P*. *aeruginosa*			
		200 μM	100 μM	50 μM	25 μM	200 μM	100 μM	50 μM	25 μM
**Di-peptide**									
1	Cys-His	64	128	256	>256	32	128	256	>256
2	Cys-Phe	32	128	128	256	16	64	128	256
5	Cys-Asp	8	16	64	128	4	16	32	128
**Tri-peptide**									
7	Cys-Gly-Phe	32	128	256	256	16	64	64	256

^a^ None of the oligopeptides possessed any antibacterial activity within the concentration range of 25–200 μM.

It was obvious that all oligopeptides existed for synergies with ertapenem at a different level. Under the same concentration of the inhibitors, the MIC values of ertapenem exhibited uniform trend towards IC_50_ values. For the di-peptides 1, 2 and 5, the difference of oligopeptides sequence was the second amino acid residue, resulting in the diversity for synergistic effect. Oligopeptide in the presence of hydrophobic side chains had a better synergistic antibacterial performance, which was consistent with the IC_50_ values against NDM-1 enzyme. At the concentrations higher than 25 μM, oligopeptide 5 was capable to sensitize the NDM-1 producing strain to ertapenem at clinically achievable concentrations (MIC ≤ 128μg/ml) [29]. The tri-peptide 7 showed the same MIC values to di-peptide 2 at the concentration of 200 μM, 100 μM and 25 μM, illustrating that the glycine in the middle of tri-peptide 7 had no contribution to improve synergies. The MIC values of ertapenem in combination with all oligopeptides at 200 μM against *E*. *coli* BL21 (DE3)/pET30a-NDM-1were higher than the MIC values against the clinical isolate of *P*. *aeruginosa*. The reason might lie in the genetically engineered bacterium of *E*. *coli* BL21 (DE3)/pET30a-NDM-1 could over-express more NDM-1 enzyme than the clinical isolate of *P*. *aeruginosa*.

### Binding model and interaction between oligopeptide and target

To accurately simulate the induced-fit effect in the enzyme-inhibitor binding process, flexible docking was performed to explore the binding model. To characterize the binding affinities of docked complexes, binding free energies (*Δ*G) calculated by MM-PBSA along with the IC_50_ values of all oligopeptides were summarized in Table 4. The binding affinity was in agreement with the previous experiment (IC_50_ values).

**Table 4:** Results of the binding free energy calculated by MM-PBSA.

Oligopeptides	Amino acid sequences	*Δ*G (kcal/mol)	IC_50_ (μM) (Mean±SD)
**Di-peptide**			
1	Cys-His	-14.6	182±14
2	Cys-Phe	-21.1	113±9
3	Cys-Tyr	-6.3	>300
4	Cys-Val	-10.2	235±18
5	Cys-Asp	-21.9	68±5
**Tri-peptide**			
6	Cys-Gly-His	-8.7	263±21
7	Cys-Gly-Phe	-16.4	144±13
8	Cys-Gly-Tyr	-4.2	>300
9	Cys-Gly-Val	-11.3	249±21
10	Cys-Gly-Asp	-4.9	>300

**Fig 2:**
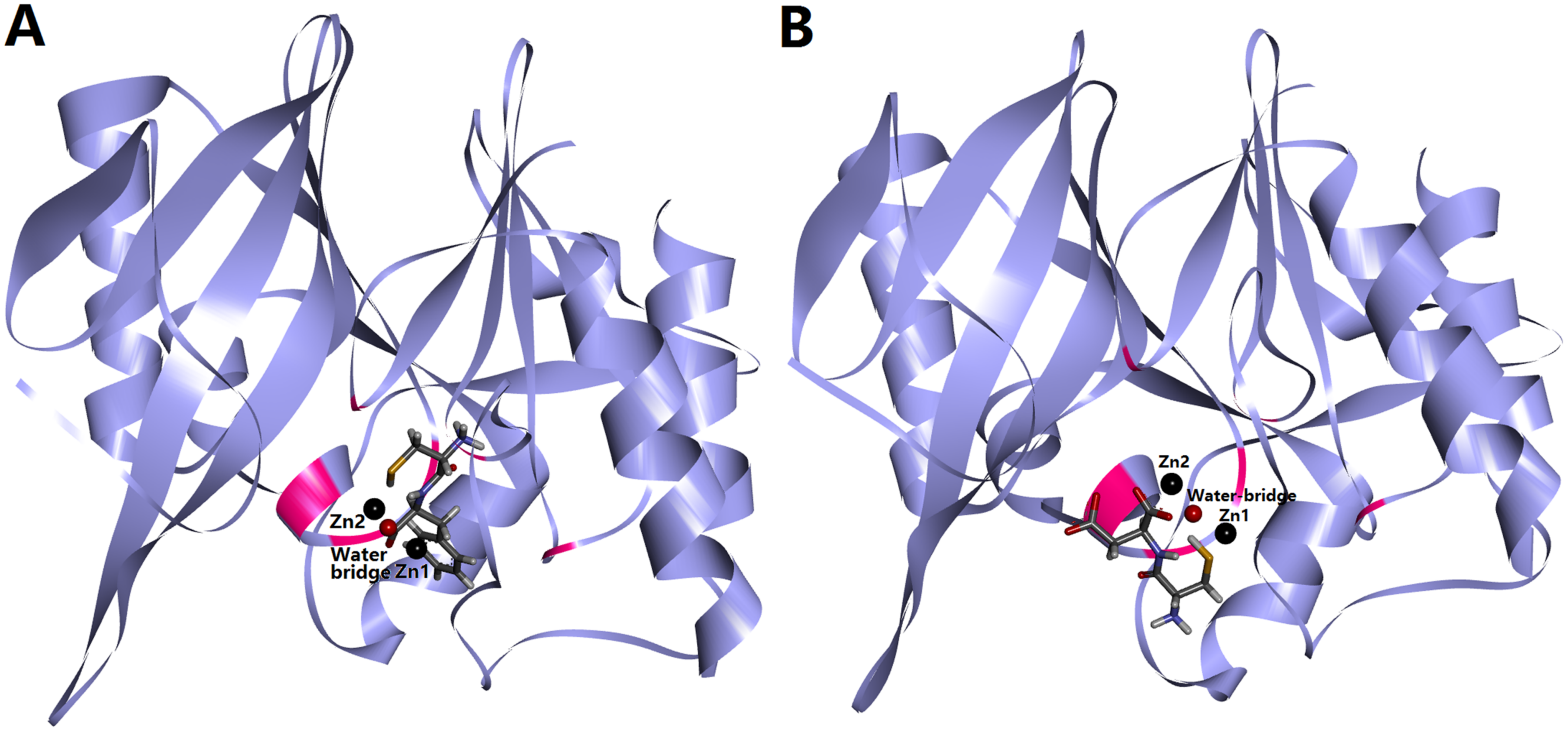
Binding model between inhibitor and target of NDM-1 enzyme. Binding models of the docked NDM-1 with oligopeptides 2 (Fig 2A) and 5 (Fig 2B), respectively. Structures of inhibitors were shown in stick model. NDM-1 was shown as ribbon model. Six key amino acids binding two zinc ions are colored by pink.

**Fig 3:**
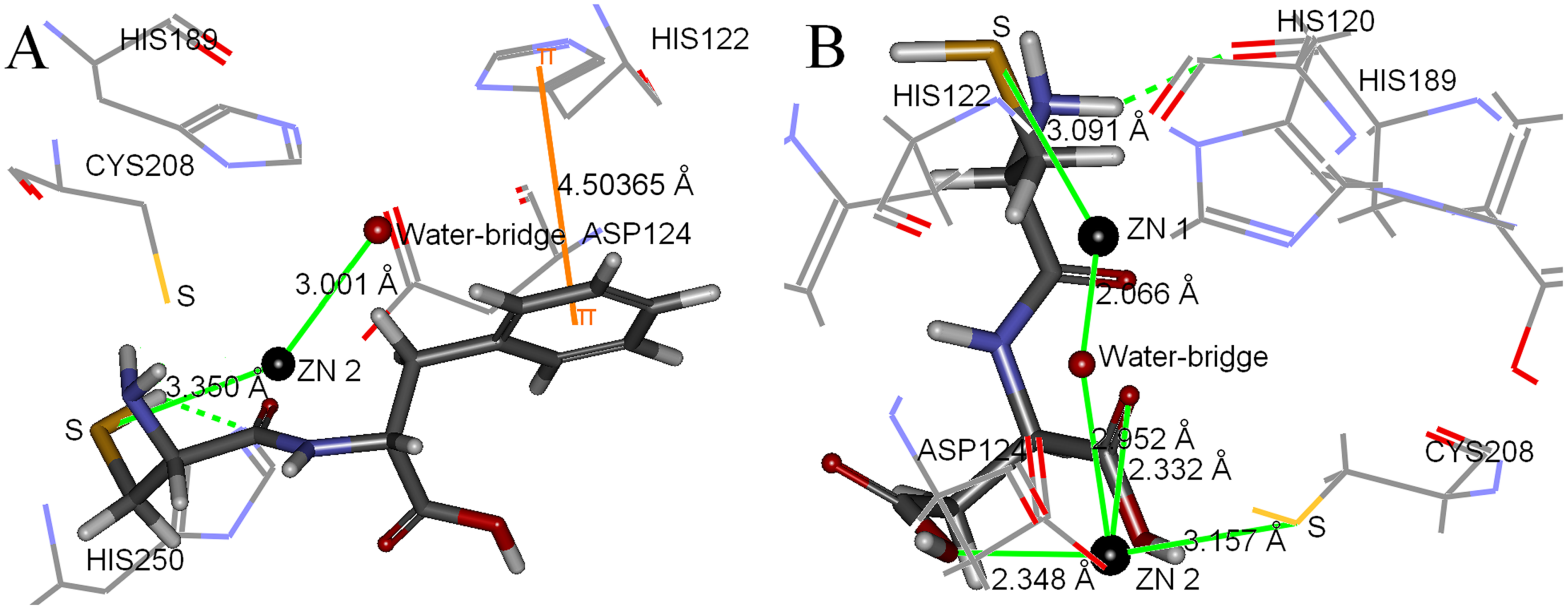
The best pose of inhibitor docked into the active site of NDM-1. Oligopeptides 2 (Fig 3A) and 5 (Fig 3B) are shown as stick model. Water-bridge and two zinc ions are shown as sphere model (red and black, respectively). Seven key amino acids in catalytic center of NDM-1 (His189, His120, His122, Asp124, Cys208, His250 and Tyr229) treated as flexible residues are shown as line model.

**Fig 4:**
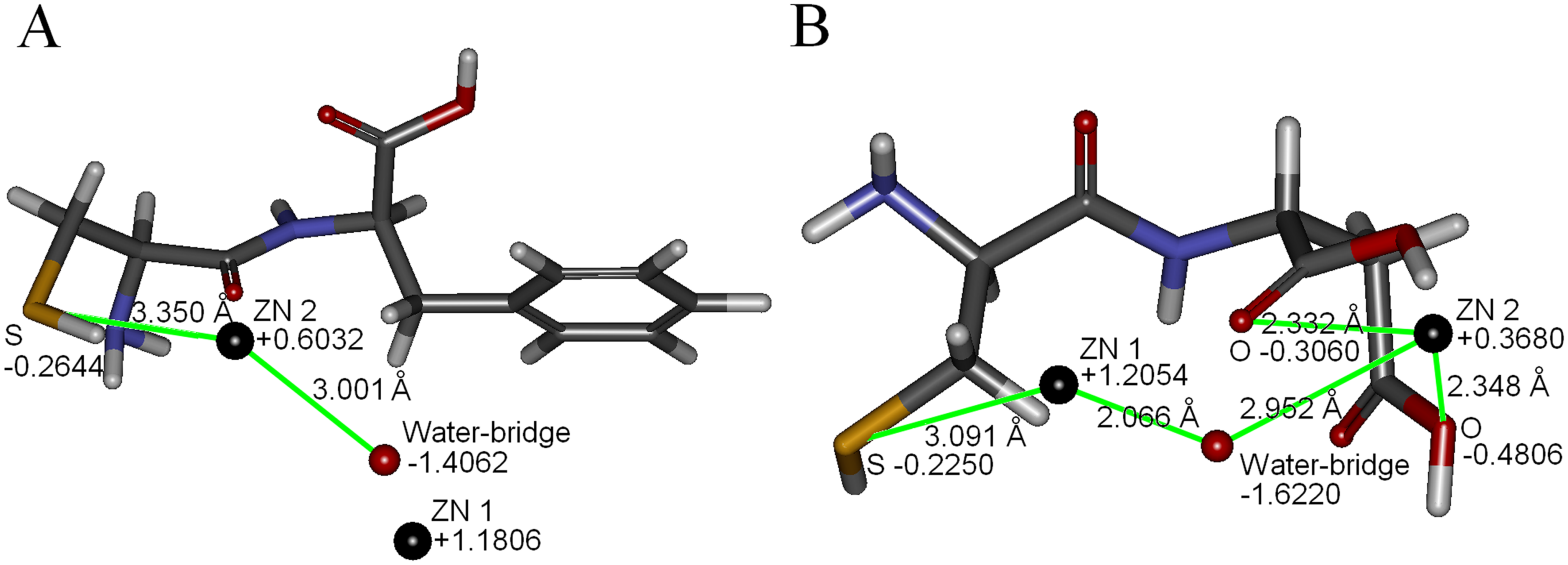
The partial charge values of water-bridge, two zinc ions, and critical atoms of inhibitors. Oligopeptides 2 (Fig 4A) and 5 (Fig 4B) are shown as stick model, which sulfur atom and oxygen atom are colored by yellow and red, respectively. Water-bridge and two zinc ions are shown as sphere model (red and black, respectively).

Two inhibitors with best activities against NDM-1, oligopeptides 2 and 5, were selected to further analyze the binding model. The binding model of oligopeptide 2 was showed in Fig 2A, suggested that the inhibitor located at the bottom of the target. The rational pose docked into the active site of NDM-1 displayed in Fig 3A. The hydrogen atom of sulfhydryl group of oligopeptide 2 and nitrogen atom of imidazole groups of His250 could form hydrogen bond. The imidazole ring of His122 was parallel to the benzene ring of oligopeptide 2, which the distance between them was 4.504Å, demonstrating these two aromatic rings exist weak π-π interaction [30]. The distance between Zn2 ion and sulfur atom sulfhydryl group of oligopeptide 2 (3.350Å) was close to the distance between Zn2 ion and water-bridge (3.001Å), indicating the electrostatic interaction might exist between sulfur atom and Zn2 ion. The results of partial charge calculation of sulfur atom and Zn2 ion were presented in Fig 4A. The accurate partial charge values of sulfur atom and Zn2 ion were -0.2644 and +0.6032, respectively. The electrostatic interaction between partial positive charge of the Zn2 ion and partial negative charge of the sulfur atom in cysteine could inhibit charge transfer in the hydrolysis process mediated by zinc ion. The π-π interaction between side chain benzyl group of phenylalanine of oligopeptide 2 and imidazole ring of His122 was able to enhance the binding strength and also restrict the conformation change of NDM-1 in the process of enzymatic hydrolysis of substrate. Hydrogen bond, π-π interaction and electrostatic effect were the three key roles for inhibitory activity of oligopeptide 2.

The binding model of oligopeptide 5 was shown in Fig 2B. The best conformation of oligopeptide 5 interacting with the key amino acids in the active site of NDM-1 was presented in Fig 3B. The hydrogen atom of cysteine group of oligopeptide 5 and oxygen atom of His120 of NDM-1 were able to form hydrogen bond. The distances between Zn2 ion and two oxygen atoms of oligopeptide 5 were within the effective range of electrostatic interactions (2.348Å and 2.332Å), since the distances between the oxygen atom of water-bridge and two zinc ions were 2.066Å (Zn1) and 2.952Å (Zn2) respectively. The distance between sulfur atom of oligopeptide 5 and Zn1 ion (3.091Å) was nearly the same as the distance between sulfur atom of Cys208 of NDM-1 and Zn2 (3.157Å), illustrating that the sulfhydryl group of oligopeptide 5 also existed weak electrostatic interaction with Zn1. Fig 4B exhibited that accurate partial charge values of sulfur atom (-0.2250), Zn1 (+1.2054), oxygen atom of water-bridge (-1.6220), Zn2 (+0.3689) and two oxygen atoms of oligopeptide 5 (-0.4806 and -0.3060) demonstrated electrostatic interactions between inhibitor and target was real existence. The sulfhydryl group of cysteine of NDM-1 and the two carboxy groups of aspartic acid of oligopeptide 5 were rich in negative charge, which had strong abilities to bind metal zinc ion. Unlike oligopeptide 2, oligopeptide 5 only had two types of interaction effects (hydrogen bond and electrostatic interaction) with the target of NDM-1. Comparing to oligopeptide 2, the stronger chelation effect caused that the oligopeptide 5 had better inhibitory activity to inactivate NDM-1 enzyme. IC_50_ value of oligopeptide 5 (IC_50_ = 68μM) was approximately one half of oligopeptide 2 (IC_50_ = 113μM).

## Conclusion

The results of inhibitory effect against NDM-1 enzyme proved that most of oligopeptides had inhibitory activities. Structure-activity relationship suggested that metal ion-binding group (such as sulfhydryl group and poly-carboxylic groups) and hydrophobic groups (such as benzyl group and iso-propyl group) were the pharmacophores significantly contributing to inhibitory activity.

The synergistic antibacterial experiments disclosed that tri-peptides comparing to the di-peptides had no advantage of synergy. The insertion of glycine led to decreasing the binding strength between inhibitor and target.

The established binding and interaction models demonstrated that hydrogen bond, electrostatic interaction and π-π interaction of parallel aromatic rings between the inhibitors and NDM-1 have played key roles in inhibiting the bioactivity of NDM-1.

The two inhibitors, oligopeptides 2 and 5, possessed the best bioactivity in all testing inhibitors. For oligopeptide 2, there are three kinds of effects (hydrogen bond, π-π interaction and electrostatic effect), while oligopeptide 5 only exists hydrogen bond and electrostatic interaction with target. We hold the opinion that if these three types of interactions achieve the optimal effectiveness, the chemical structure modification product of oligopeptides 2 and 5 may bring us an exciting bioactivity. Now, further chemical modifications of oligopeptide 2 are underway.
